# Deaf-Mutism and Consanguineous Marriage

**Published:** 1887-12

**Authors:** Charles Warden

**Affiliations:** M. D., F. R. C. S. ED., Senior Honorary Surgeon to the Royal Institution for the Education of Deaf and Dumb Children, Birmingham, and to the Birmingham Ear and Throat Hospital


### DEAF-MUTISM AND CONSANGUINEOUS MARRIAGE.

BY CHARLES WARDEN, M. D.. F. R. C. S. ED.,

Senior Honorary Surgeon to the Royal Institution for the Education of Deaf and Dumb Children, Birmingham, and to the Birmingham Ear and Throat Hospital.

Being connected with the Royal Institution for the Education of Deaf and Dumb Children in Birmingham, I thought it possible that I might lay before the Association some important facts and statistics which would be of interest to the members. This institution has been established upwards of seventy-four years, and is the oldest but one in England; although it is known as a Birmingham charity, its area of usefulness extends all over England. The number of children in the institution is 136—boys 75, girls 61; and there are now at least fifty in the town waiting their turn to be admitted. Additions are being made to the institution, and it is hoped the accommodation will then meet that requirement.

The condition of an uneducated deaf-mute is, indeed, a sad one. He is cut off from all familiar sounds; shut out from all participation in the intelligence of the world ; excluded from the greater part of the pleasures and pursuits of life. What is more awful still, the social and intellectual degradation of uneducated deaf-mutes is thrown into the shade by the darkness of their spiritual state. The deaf and dumb have intellectual faculties, especially the faculties of observation, memory and comparison, and under education these faculties become capable of great development.

It is a great satisfaction to learn of the appointment of a Royal Commission to inquire into the condition and means for the education of the deaf and dumb and the blind, and it is hoped that its labors will result in state aid to existing establishments engaged in the education of these classes.

The number of males exceeds that of females in deaf-mutes—that is, in the civilized world. In European countries Netherlands bears the smallest proportion (three mutes to 10,000); Switzerland the greatest (Berne, 54 to 10,000); entire average, 24 to 10,000; in some districts, 50 to 10,000); Belgium, 4 to 10,000; Great Britain, 5 to 10,000; Denmark, 6 to 10,000; France, 6 to 10,000; Spain, 6 to 10,000; Italy, 7 to 10,000. United States are below the average. Above averages are Germany, 9 to 10,000; Austria, 9 to 10,000; Hungary, 13 to 10,000; Sweeden, 10 to 10,000; Norway, 9 to 10,000. In German States the number of deaf mutes is larger than that of blind; whereas, in most other countries blindness occurs more frequently than deaf-mutism. In Switzerland the number of deaf-mutes is three times that of the blind; there are more deaf-mutes in mountainous districts, more blind in lowlands. In Bavaria, where infant morality is exceedingly high, there are few deaf-mutes; in parts where it is moderate deaf-mutes are numerous—accounted for, possibly, by earlier deaths.

The deaf-mutes of Martha's Vineyard, who have attracted considerable attention in America, come chiefly from the town of Chilmark, containing a population of 500, of whom twenty are deaf-mutes, one in 25. The cause of the prevalence is not established beyond question; the probability is they are descended from common ancestors. The facts obviously illustrate the tendency of deafness to perpetuate itself in certain families, and the increased intensity of this tendency when members of those families marry one another.

The Spartans doomed all deaf and dumb children to death, and compelled their parents to cast them into the fœtid caverns; the Romans looked upon them as the scorn of mankind. The Justinian code condemned them to one ignominious state of imbecility.

It is very probable that the work published in 1620 — upon which the existing French and American systems of deaf-mute education are based — originated with a Benedictine monk in Spain, in the sixteenth century, named Pedro Ponce, who was the first practical teacher, and was modified by Bonet. In England the first to call attention to the subject was John Bulwer, in 1648.

The learned Bede, who died A. D. 735, recorded a case of deaf-dumbness in a youth, who had been taught to speak; then Rudolph Agricola, who died in 1485, had an idea of the possibility of teaching the deaf and dumb.

Deafness is not strictly a hereditary disease, though parents do have deaf children; when parents are both innately deaf it is extremely probable that their offspring will be naturally deaf; and if the children marry others naturally deaf, their offspring will be similarly afflicted; but if the deafness has arisen from an accidental cause, and there is no consanguinity or struma, then probably all their children will be perfect. Sometimes alternate generations will be affected; at others two generations are skipped, and it appears in a later one. The proportion is 80 per cent. congenital to 20 per cent. acquired.

When a number of children are born deaf in one family, heredity plays a part in its production; in such cases one must look for the causes in ancestry.

In regard to the second query, it is reasonable to assume that in the ancestors of a large number of unrelated deaf-mutes, light might be thrown upon the origin of deafness by a comparison of genealogies. Boudin especially considered that the marriage of blood-relatives favors the occurrence of deaf-mutism, blindness, mental diseases and great mortality amongst children. In Paris it produced 28 per cent., and in Bordeaux Institution one-third. Perrin found 25 per cent. in Lyons.

The consanguinity of parents (first cousins) is a cause. Two twin brothers married two sisters (cousin german); the elder had several children, one deaf and dumb; of the younger six children, three can hear, three cannot.

Fright and strong mental morbid impressions on the part of the mother during pregnancy are among the physiological causes.

Organic defects is a pathological cause. In my own practice at the Birmingham Ear and Throat Hospital, and also at the Orthepædic and Spinal Hospital, I have had numerous instances of the effect of mind upon matter in producing deaf-mutes, and especially of congenital deformities.

*Organic Defect, the Chief and True Pathological Cause.*— In every case of congenital deafness the deficiency is owing to some lesion, either in the mechanical or sensitive portion of the auditory apparatus of the brain. The abnormal conditions are total deficiency of the external auditory meatus, adventitious growths filling up the cavity of the tympanum, the Eustachian tubes, mastoid cells and other foramina of the tympanum, absence of ossicula or ankylosis of them, deficiency or enlargement of the fenestra rotunda, spiral canals of the cochlea or aqueduct of the vestibule; obliteration of semicircular canals; preternatural density of petrous portion of temporal bone; defects of the labyrinth; atrophy of auditory nerve. Dr. Itard considered that nerve-deafness arose from paralysis of the labyrinthine nerve. Mr. Pilcher, whose pupil I had the honor, pleasure and satisfaction of being, taught that, in a majority of cases he examined, the cause is not demonstrated to account for the loss of function. Postnatal deafness arises where inflammation, ulceration or paralysis accompany or supervene upon diseases, such as sacarlatina, typhus, small-pox, measles or convulsions—more from sacarlatina than any other; and, last, also diphtheria.

*Other Causes.*— Then while congenital deafness is principally caused by transmission from parents, under circumstances not yet fully understood, acquired deafness is brought about by diseases which destroy the organs intended for the perception of sound. The great majority of these diseases do not originate in the organ of hearing, but the deafness in most cases is caused by inflammation of the brain and its membranes, as also by general diseases which affect the ear—in which case the inflammation seems to affect the auditory nerve and labyrinth.

After brain disease, scarlet fever ranks second among the causes of acquired deafness; other zymotic diseases, third; injuries, etc., fourth.

Cerebro-spinal meningitis is another fertile source. In Prussia 278 deaf-mutes were produced by it. Erhardt was of opinion that the diseases produce hæmorrhage in the labyrinth, but this assumption is not borne out by *post-mortem* examinations.

In chronic inflammation of labyrinth hyperæmic swelling, fatty degeneration, atrophy of the membranous labyrinth, changes and deposits in the labyrinthine fluid take place. Naso-pharyngeal catarrhs are almost common causes of deafness.

Our knowledge of the anatomical changes in deaf-mutism are inexact and incomplete. We have no *post-mortem* records of deaf-mutes that were the offspring of consanguineous marriages, or who had inherited the defect; it would be of the greatest importance to learn on what organic conditions these causes depend. They are classed as malformations, anatomical abnormalities in the middle ear, in the labyrinth and auditory nerve, and in the brain.

*Deafness and Blindness.*—Prussia (1871 census) showed 201 deaf-mutes and blind out of 23,000. In Bavaria, in 4,348 deaf mutes there were 23 blind. In the year 1840, in Sweeden, in 2,100 deaf-mutes there were 90 blind. Diseases of the eye not unfrequently accompany those of the ear, especially in strumous constitutions. The exanthemata, small-pox especially, cause affections of both eye and ear simultaneously.

Rentinitis pigmentosa is either congenital or occurs in childhood. Hereditary predisposition and the blood relationship of the parents are the principal causes of its occurrence. According to Leber, these causes can be traced in one-half the cases, hereditary predisposition being the cause as frequently as consanguinity of parents. Liebreich was the first to draw attention to the simultaneous occurrence of retinitis pigmentosa and deaf-mutism, and from his investigations come the following conclusions:

1. That retinitis pigmentosa occurs very frequently in deaf-mutes

2. That a simultaneous occurrence of both defects is chiefly observed in Jewish children (Liebreich was a Jew).

3. That in the majority of the children thus affected, the defects are due to the consanguinity of the parents. Liebreich examined 241 deaf-mutes, and found 14 with retinitis pigmentosa. The result of only one *post mortem* examination of a case of deafness associated with retinitis pigmentosa was reported by Lucæ, in which large quantities of calcareous concretions were discovered in both vestibules, and both vestibules were partially sclerosed, probably the remains of inflammation of the labyrinth in early childhood.

4. Cerebro-spinal menengitis is also a fertile cause of deafness. Of 77 pupils in the Pennsylvania Institution, 66 were cases of adventitous deafness, 18 having lost the hearing by cerebro-spinal meningitis. In Lackawana 85 per cent. are caused by this disease.

With regard to blood relationship of parents, Devay mentions an instance observed by Menière. Married cousins had eight children: four deaf-mutes, one idiotic, one died of encephalitis, two perfectly deaf. Mitchell computes that, if a deaf-mute marries a healthy person, the chances of a deaf-mute offspring are as 1 to 135; if two deaf-mutes marry, 1 to 20. If a deaf-mute marries a healthy cousin, he is in the same danger as if he had married a deaf-mute outside his family. If the cousin he has married is also a deaf-mute, the union is most dangerous.

Alexander Graham Bell has written a memoir on the formation of a deaf variety of the human race. Dr. Bell does not advocate legislative interference with the marriage of the deaf-mute, for several reasons, one of which is that the results of such marriages have not yet been sufficiently investigated. While Dr. Bell's statistics do not establish the probability of the formation of a deaf variety of the human race, they certainly prove that heredity is a very important element in the cause of deaf-mutism. The principle of heredity is clearly proved by the large number of deaf mutes who are related by blood to each other; 295 in 1,000 had one or more deaf-mute relatives in six American schools. Dr. Bell gives statistics of some families in the United States that have become famous in the annals of deaf-mutism for the large number of deaf-mutes they contain — the Browns of New Hampshire having deaf-mutes in four consecutive generations, and numbering 34 such cases; the Hoaglands of Kentucky containing 21 deaf-mutes in three consecutive generations; and a group of ten families in Maine, not known to be connected, contain 105 deaf-mutes. Dr. Bell's statistics show that many married deaf-mutes have no deaf-mute children, but they also show that the proportion of deaf children with deaf parents is far greater than with hearing parents.

Another curious fact shown by Dr. Bell's statistics is that the proportion of deaf mute children is greater when one of the parents is deaf and one hearing than when both parents are deaf. Although the statistics of heredity are too limited and incomplete to form positive conclusions, the following seem probable:

1. Deaf-mute relatives marrying deaf-mute relatives are likely to have deaf-mute children.

2. Congenital deaf mutes marrying each other, especially if either have deaf-mute relatives, are likely to have deaf-mute children.

3. The adventitiously deaf, and not having deaf-mute relatives, marrying each other, are not likely to have deaf-mute children.

4. Deaf-mutes, whether congenital or adventitious, not having deaf-mute relatives, and marrying hearing persons who have not deaf-mute relatives, are not likely to have deaf-mute children.

In conclusion, it appears self-evident to my mind that segregation is the remedy for anything approaching this condition.—*British Medical Journal.*

